# Modified Clonidine Testing for Growth Hormone Stimulation Reveals α_2_-Adrenoreceptor Sub Sensitivity in Children with Idiopathic Growth Hormone Deficiency

**DOI:** 10.1371/journal.pone.0137643

**Published:** 2015-09-11

**Authors:** Christian Willaschek, Sebastian Meint, Klaus Rager, Reiner Buchhorn

**Affiliations:** Caritas Krankenhaus, Department of Pediatrics, Bad Mergentheim, Germany; TNO, NETHERLANDS

## Abstract

**Introduction:**

The association between short stature and increased risk of ischemic heart disease has been subject to studies for decades. The recent discussion of cardiovascular risk during growth hormone therapy has given new importance to this question. We have hypothesized that the autonomic system is a crucial element relating to this subject.

**Methods:**

Heart rate variability calculated from 24-hour electrocardiogram data is providing insight into the regulatory state of the autonomous nervous system and is an approved surrogate parameter for estimating cardiovascular risk. We have calculated heart rate variability during clonidine testing for growth hormone stimulation of 56 children. As clonidine is a well-known effector of the autonomous system, stimulating vagal tone and decreasing sympathetic activity, we compared the autonomous reactions of children with constitutional growth delay (CGD), growth hormone deficiency (GHD) and former small for gestational age (SGA).

**Results:**

During clonidine testing children with CGD showed the expected α_2_-adrenoreceptor mediated autonomous response of vagal stimulation for several hours. This vagal reaction was significantly reduced in the SGA group and nearly non- existent in the GHD group.

**Discussion:**

Children with GHD show a reduced autonomous response to clonidine indicating α_2_-adrenoreceptor sub sensitivity. This can be found prior to the start of growth hormone treatment. Since reduction of HRV is an approved surrogate parameter, increased cardiovascular risk has to be assumed for patients with GHD. In the SGA group a similar but less severe reduction of the autonomous response to clonidine was found. These findings may enrich the interpretation of the data on growth hormone therapy, which are being collected by the SAGhE study group.

## Introduction

Growth is considered to be the best global indicator of child’s well-being. Growth impairment has both short and long-term consequences. Progressive decline in nutritional status is linked with active and poorly controlled disease. There is strong evidence that poor growth is associated with delayed mental development and that there is a relationship between impaired growth status and both poor school performance and reduced intellectual achievement [1,2]. Moreover, the inverse association between height and ischemic heart disease has been studied extensively over the last six decades, from the first report in 1951 to a recent meta-analysis of 52 studies [3]. In summary adults in the shortest height category were at approximately 50% higher risk of ischemic heart disease related morbidity and mortality than tall individuals. Mainly patients with low birth weight (in the stricter sense “small for gestational age” = SGA) [4] and growth hormone deficiency (= GHD) [5] are in the focus of current investigations. If these children are candidates for growth hormone therapy then the effect of this therapy on cardiovascular risk has to be clarified. Autonomic imbalance may be a final common pathway to increased morbidity and mortality in cardiovascular disease. Global heart rate variability (HRV) indicated by the parameter SDNN (standard deviation of normal RR-intervals) may be used to assess autonomic imbalances, diseases and mortality risk in adults [6,7] and children [8]. Sympathetic activity indicated by low rMSSD (root mean square standard deviation of adjacent RR intervals) and pNN50 (Percentage of normal to normal intervals differing more than 50 msec. from the proceeding interval) has been associated with a wide range of conditions including cardiovascular disease. Recently a reduced response to the clonidine growth hormone stimulation test was found to be associated with unfavourable cardiovascular risk factor profile in childhood cancer survivors [9].

The α_2_-adrenoreceptor agonist clonidine (2-[(2,6-Dichlorphenyl) imino]imidazolidin) is a powerful inhibitor of the sympathetic autonomic system. It stimulates growth hormone (GH) release in both children and adults. The substance is used to test for GH deficiency in children. In adults it has been used to assess central α_2_-adrenoreceptor function in order to determine the pathophysiological basis and to confirm the diagnosis of neurological diseases associated with autonomic failure. The secretion of growth hormone is regulated by a complex neuroendocrine control system based on the balance between two hypothalamic neuro hormones: growth hormone-releasing hormone, exerting a stimulating influence upon growth hormone secretion and somatostatin, which has an inhibitory effect. The release of growth hormone-releasing hormone and somatostatin is modulated by neurotransmitters, such as norepinephrine and acetylcholine. Specifically the activation of hypothalamic α_2_-adrenoceptors and muscarinic cholinergic receptors induces growth hormone release by stimulating growth hormone-releasing hormone and by inhibiting somatostatin. In summary clonidine effect on growth hormone release is based on its action upon α_2_-adrenoceptors.

For a better understanding of the link between human height and cardiovascular risk we analysed HRV in children with a height below the third percentile. We investigated HRV during a routine clonidine growth hormone stimulation test (N = 56) and compared vagal response of patients diagnosed with GHD, SGA and CGD with a control group of healthy children within the normal height range.

## Methods and Ethical Statement

### Control group

Normal healthy children (group 1, N = 56) were collected from our “normal values heart rate variability project”. Therefore data of patients, who attended our outpatient clinic for exclusion of cardiac arrhythmia were analysed retrospectively. The retrospective analysis was approved by the ethical board of our county medical chamber (Landesärztekammer Baden Württemberg) and recently published [10]. No further written consent was required. Children with a body mass index above 96% and height below 3% were excluded from the control group. Data were anonymized prior to further statistical analysis. As the controls were healthy, they did not undergo Clonidine stimulation or laboratory testing for growth hormone deficiency.

### Patients

Between 2010 and 2013 patients, who attended our clinic with short stature below the third percentile, have been included in our research. Clonidine testing was performed as a clinical standard procedure in order to diagnose or exclude growth hormone deficiency. Since clonidine testing is routinely recommended for children with diagnosed growth failure, no further written consent was required. 24-hour electrocardiograms were performed to increase patient safety during clonidine testing. Patients with acute illness were not suitable for clonidine testing. The clonidine group consists of 36 children with CGD (group 2), 14 with GHD (group 3) and 6 with SGA (group 4).

### Clonidine testing

Patients with short stature and suspected growth hormone deficiency spent a day at our clinic for a clonidine test. Clonidine stimulation was performed with an oral dose of 0.15 mg/m^2^ body surface area. Blood samples for growth hormone measurements were taken 15 minutes before and 45, 60, 90 and 120 minutes after clonidine application [11,12]. After clonidine application patients were monitored closely for heart rate and blood pressure. Transient arterial hypotension was treated appropriately with 10 ml/kg body weight normal saline bolus therapy. For improvement of patients’ safety 24-hour ECG were additionally performed.

### 24-hour ECG and analysis of heart rate variability

Measurement and interpretation of HRV parameters in the current sample were standardized according to the Task Force Guidelines [13]. Cardiac autonomic functioning was measured by 24-hour Holter 12 bit digital ECG (Reynolds Pathfinder II, Spacelabs, Germany; 1024 scans/sec). Daytime and night-time periods were defined according to patient protocols. All Holter recordings were reviewed by the same experienced cardiologists (RB and CW) and were edited to validate the systems QRS labelling. Measures of HRV were calculated employing only normal-to-normal intervals. QRS-complexes classified as noise were excluded from the data. A minimum of 23 hours of analysable data and minimally 95% of analysable NN intervals were required for the data to be included. For time domain measures, mean RR interval, resulting heart rate and the following HRV parameters were calculated as average hourly values and as 24-hour average values: square root of the mean of the sum of squares of differences between adjacent NN-intervals (rMSSD) and the standard deviation of NN intervals (SDNN). rMSSD reflects predominantly changes in vagal tone. SDNN is a parameter which reflects global heart rate variability. It is dually influenced by cholinergic and adrenergic activity, as well as other physiological inputs. In the following we use the term vagal tone for the part of the ANS measured by rMSSD. For frequency domain measures, beat-to-beat fluctuations were transformed to the frequency domain using Fast Fourier Transformation. Spectral power was determined over three frequency regions of interest: Very low frequency (VLF, < 0.04 Hz), low frequency (LF, 0.04–0.15 Hz) and high frequency (HF, 0.15–0.4 Hz) with derived HF/LF ratio. LF reflects mostly sympathetic activity, whereas HF is a reflection of vagal tone.

### Statistical analysis

Statistical analysis was performed with SPSS version 21 (IBM).

Since heart rate variability is an age related parameter, normal value controls for each group have been matched according to age and sex.

Groups were compared with controls by means, standard deviation and t-testing.

Patients and control groups were compared using non parametric t-testing. P-values below 0.05 were considered as statistically significant.

The hypothesis of correlation of data was estimated by calculating Spearman’s rho.

From anthropometrical data percentile values, SDS_LMS_ scores and BMI have been calculated using the free myBMI web page (now changed to myBMI4Kids) according to percentile data by Kromeyer-Hauschild et al. [14].

## Results

### 24-hour analysis

Groups did not differ significantly in age or BMI (Table 1). All children undergoing stimulation testing were significantly smaller than controls (p < .001). On the day of clonidine testing the 24-hour heart rate was lower in the testing groups (group2, 3 and 4) compared with the controls, who did not receive clonidine. Lower heart rate was only significant for CGD (p < .001). Only the CGD group (group 2) showed significant decrease of normalised LF (p < .05) and increased HF/LF ratio during daytime (p < .001) (Table 2).

**Table 1:** Anthropomethrical data of healthy controls and children with CGD, GHD and SGA.

		Clonidine Test (Age matched T-Tests)		
Parameter	Healthy Control	Constitutional Growth Delay	Growth Hormone Deficiancy	Small for Gestational Age
**N**	56	36	14	6
**Age [years]**	8.9 ± 4.4	6.9 ± 2.7	7.8 ± 3.3	5.0 ± 1.1
**Birthweight [gram]**		3187 ± 535	3127 ± 789	1701 ± 756****
**Gestational Age [weeks]**		38.9 ± 1.8	39.8 ± 4.1	35.0 ± 5.5**
**Height [centimeter]**	123 ± 19	108 ± 16***	109 ± 16***	99.6 ± 7.0***
**Height Percentile [%]**	52 ± 28	3,1 ± 5,4	3,5 ± 4,4	2,8 ± 4,6
**Height (SDS**_**LMS**_ **)**	0.1 ± 0.9	-2.1 ± 0.6	-2.2 ± 0.7	-2.3 ± 1.5
**Weight [kilogram]**	26.1 ± 13	18.7 ± 7.3***	18.3 ± 7.1	14.1 ± 0.4
**Weight Percentile [%]**	48 ± 28	7 ± 10	11 ± 16	1,8 ± 2,1**
**Weight (SDS**_**LMS**_ **)**	± 1.0	-1.8 ± 0.7	-1.8 ± 1.0	-2.6 ± 1.1
**BMI**	16.3 ± 3.5	15.2 ± 1.4	15.7 ± 2.3	14.3 ± 1.2
**BMI Percentile [%]**	45 ± 31	34.3± 25	34 ± 26	25 ± 26
**BMI (SDS**_**LMS**_ **)**	-0.1 ± 1.1	-0.5 ± 0.8	-0.6 ± 1.0	-0.9 ± 1.0

BMI: Body mass index, SDS_LMS_: Standard deviation score for not normally distributed data.

**** p < 0.0001;

*** p < 0.001;

** p < 0.01.

**Table 2:** Growth factors and HRV data of healthy controls and children with CGD, GHD and SGA.

		Clonidine Test (Age matched T-Tests)		
Parameter	Healthy Control	Constitutional Growth Delay	Growth Hormone Deficiency	Small for Gestational Age
**N**	56	36	14	6
**IgF1 [100–476 ng/ml]**	90 ± 15	86 ± 56	76 ± 42	43 ± 30
**IgFPB3 [1900–5200 μg/l]**	149 ± 51	3075 ± 1076	2863 ± 1254	2538 ± 644
**NT-pro-BNP [<289 ng/l]**	46 ± 18	162 ± 169	178 ± 172	177 ± 94
**Heart Rate [bpm]**	34 ± 15	85 ± 11***	85 ± 11	83 ± 8*
**SDNN [ms]**	61 ± 26	112 ± 27**	117 ± 33*	142 ± 40
**RMSSD[ms]**	4957 ± 3029	51 ± 19*	50 ± 17	77 ± 15**
**RMSSD Day**	3889 ± 2747	41 ± 16**	37 ± 16	53 ± 14**
**RMSSD Night**	6287 ± 3843	65 ± 24	70 ± 24	108 ± 24**
**Total Power**	2839 ± 2222	3992 ± 2082	4000 ± 1873	7298 ± 2580**
**Total Power Day**	2256 ± 2016	3048 ± 1629	3286 ± 1863	4720 ± 1971*
**Total Power Night**	3534 ± 2844	5289 ± 3118	5111 ± 2391	10740 ± 3264**
**VLF**	60 ± 8	1960 ± 1327	2026 ± 1085	4831 ± 2678*
**VLF Day**	65 ± 9	743 ± 124	1666 ± 947	2710 ± 1601
**VLF Night**	56 ± 10,6	2699 ± 2412	2581 ± 1595	7553 ± 3833*
**LFn**	731 ± 350	52 ± 10*	55 ± 10	65 ± 12
**LFn Day**	456 ± 271	53 ± 9***	59 ± 11	61 ± 12
**LFn Night**	1100 ± 556	51 ± 14	53 ± 12	70,8 ± 13
**HF**	0.61 ± 0.22	817 ± 334	759 ± 295	747 ± 376
**HF Day**	0,45 ± 0,18	636 ± 371**	553 ± 331	633 ± 268*
**HF Night**	0,79 ± 0,38	1082 ± 451	1079 ± 483	945 ± 671
**HF/LF**	90 ± 15	0,85 ± 0,36*	0,78 ± 0,32	0,51 ± 0,33
**HF/LF Day**	149 ± 51	0,77 ± 0,34***	0,65 ± 0,32	0,59 ± 0,40
**HF/LF Night**	46 ± 18	0,99 ± 0,55	0,95 ± 0,66	0,43 ± 0,30*

Laboratory data, data on 24- hour HRV, daytime und night time HRV from children with CGD, GHD and SGA compared to age-matched healthy controls.**IgF1:** Insuline like growth factor, **IgFPB3:** Insuline like growth factor binding proteine, **NT-pro-BNP:** N-terminal propeptide of brain natriuretic peptide, **SDNN:** Standard deviation of NN-intervalls, **RMSSD:** Root mean square standard deviation of adjacent NN Intervals, **VLF:** Very low frequency (< 0.04 Hz),**LF:** low frequency (LF, 0.04–0.15 Hz) **HF:** high frequency (HF, 0.15–0.4 Hz).

*** p < 0.001;

** p < 0.01;

* p < 0.5.

#### Clonidine effect

**Fig 1:**
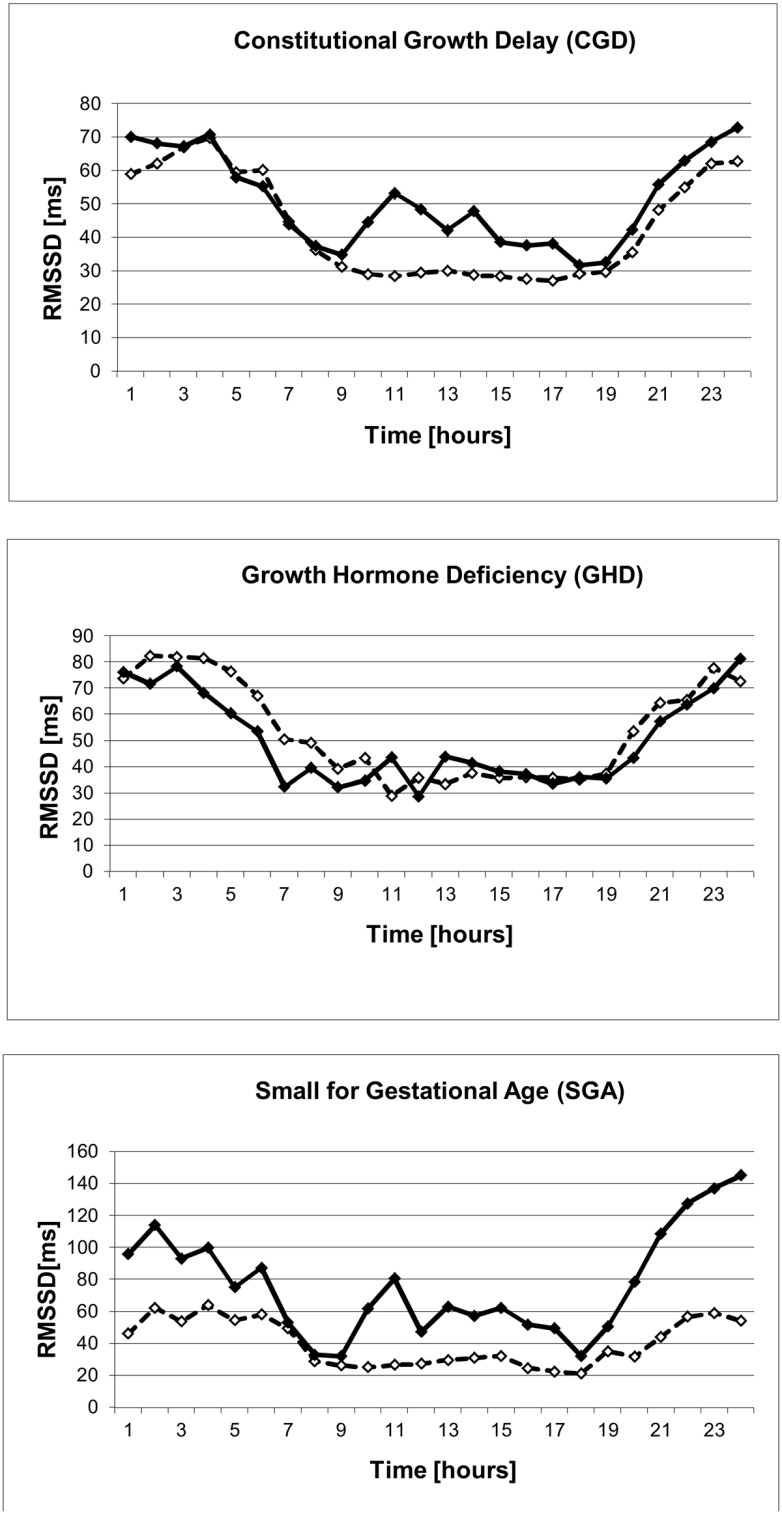
Effect of clonidine on rMSSD in normal and short stature children. Effect of clonidine given at 9:00 a.m. on the vagus parameter rMSSD in children with constitutional growth delay (n = 36) (♦) compared to baseline data of age-matched healthy controls (◊). Children with growth hormone deficiency (n = 14) and small for gestational age (n = 6) show reduced vagal activation after clonidine administration (♦).

**Fig 2:**
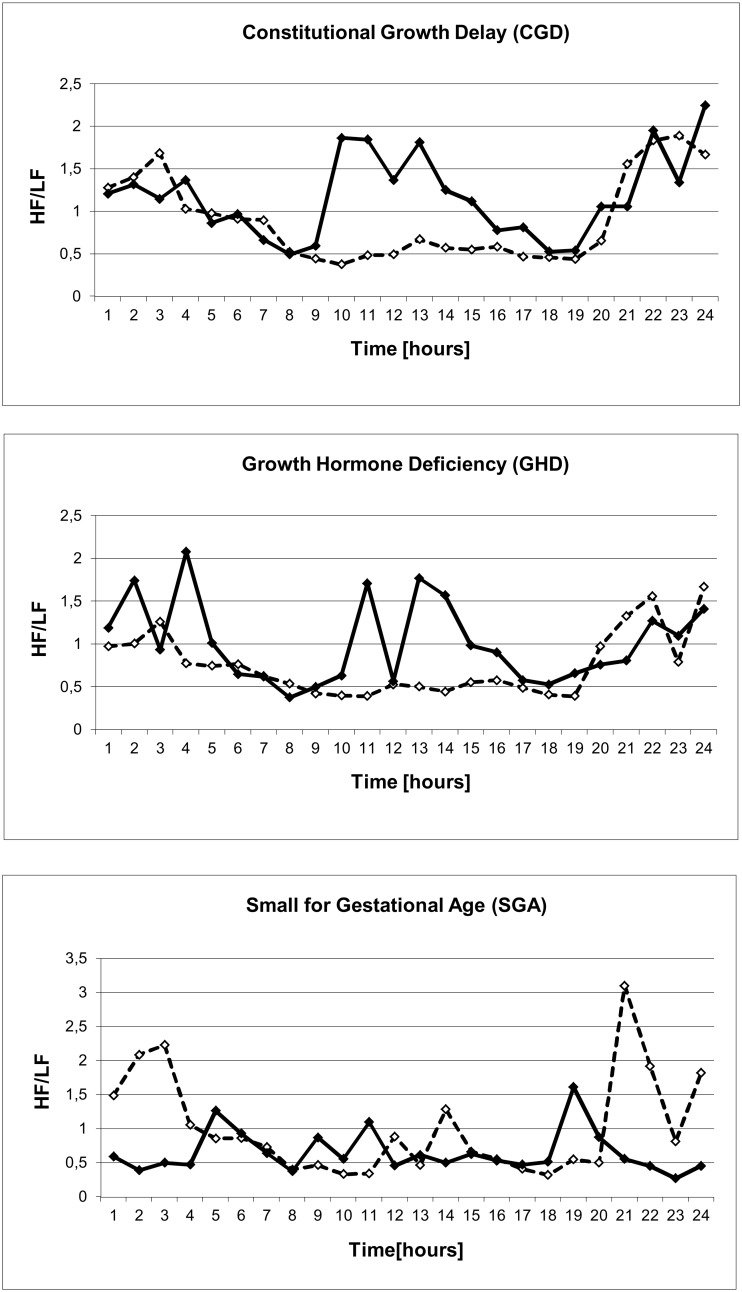
Effect of clonidine on HF/LF in normal and short stature children. Effect of clonidine given at 9:00 a.m. on the autonomic balance indicated by the parameter HF/LF compared to baseline data of age-matched healthy controls(◊). Children with constitutional growth delay (n = 36) show a clear rise of HF/LF ratio indicating sympathetic inhibition (♦). The effect is smaller in growth hormone deficient children (n = 14) and absent in children small for gestational age (n = 6) (♦).

**Fig 3:**
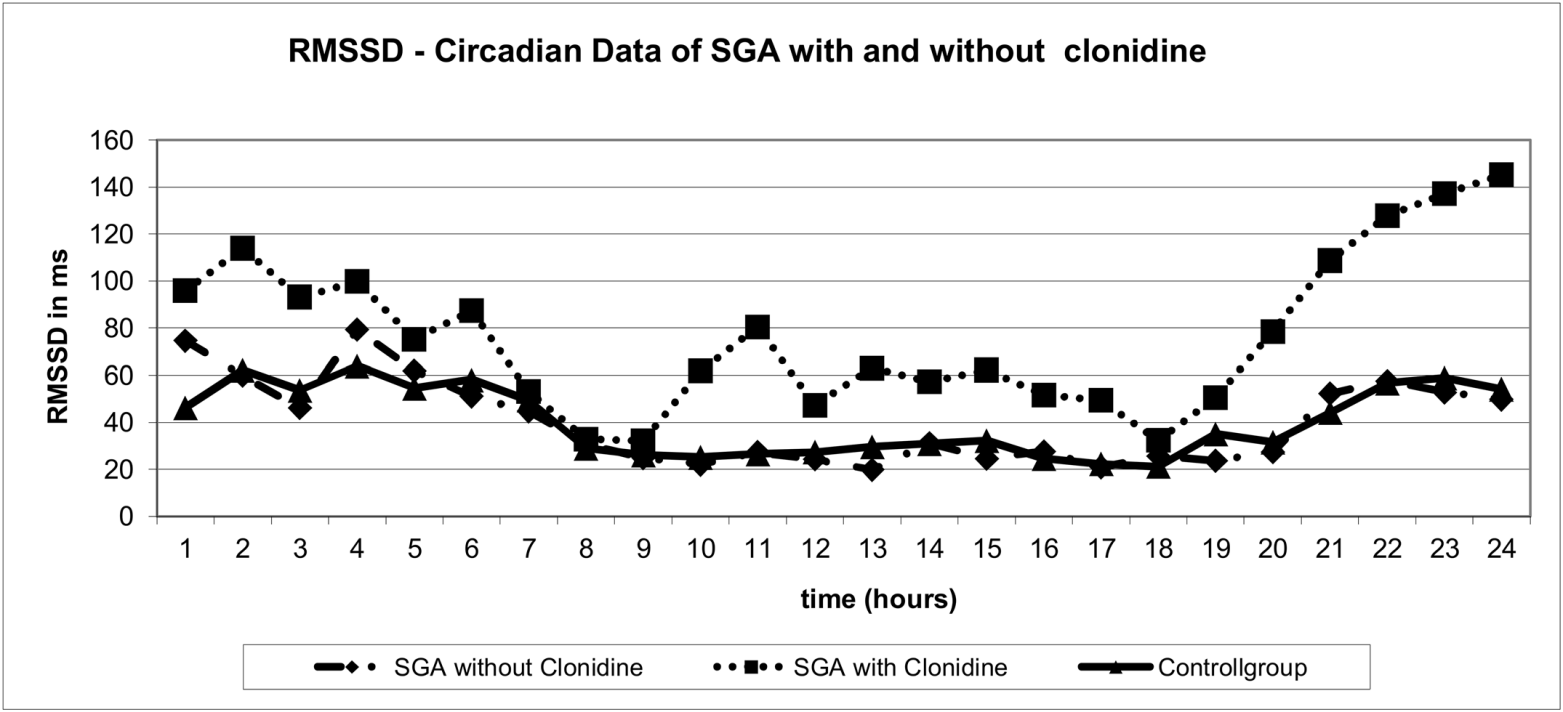
rMSSD-Circadian Data of SGA with and without clonidine. Effect of clonidine given at 9:00 a.m. on the vagus parameter rMSSD in children with SGA (n = 6) after clonidine administration (■) compared to age-matched SGA without clonidine (♦) and to age-matched healthy controls (▲).

As expected clonidine showed an overall sympatholytic effect indicated by lower LF and higher HF/LF values and led to vagal activation indicated by higher rMSSD at daytime for about six hours (Table 3, Table 4, Figs 1 and 2). However during clonidine testing we observed vagal activation as rMSSD only in CGD (p < .001 at 10:00 and 11:00) and SGA children (p .015 and .012 at 10:00 and 11:00 o'clock) (Table 3). No vagal activation could be measured in the GHD group. There seem to be higher rMSSD values at night in the SGA group independent of clonidine given at 9:00 in the morning (Table 3, Figs 1 and 3). In contrast sympathetic inhibition by clonidine indicated by higher HF/LF was only observed in children with CGD and GHD, but not in children with SGA (Fig 2, Table 4).

**Table 3:** rMSSD values during clonidine testing and healthy controls.

	Baseline		rMSSD [ms] during Clonidine Test started at 9:00 T- test versus age matched baseline controls								
	Healthy Control		Constitutional Growth Delay			Growth Hormone Deficiency			Small for Gestational Age		
	N = 56		N = 36			N = 14			N = 6		
Time	MW	SD	MW	SD	p-value	MW	SD	p-value	MW	SD	p-value
**8**	**36.2**	**20.7**	**37.4**	**19.1**	**0.80**	**39.5**	**27.4**	**0.43**	**33.0**	**12.7**	**0.644**
**9**	**31.2**	**17.8**	**34.9**	**20.0**	**0.42**	**32.2**	**18.7**	**0.52**	**32.1**	**11.8**	**0.421**
**10**	**29.0**	**15.5**	**44.7**	**19.2**	**<0.001**	**34.8**	**18.4**	**0.29**	**61.9**	**26.0**	**0.015**
**11**	**28.5**	**18.3**	**53.3**	**23.5**	**<0.001**	**43.5**	**25.9**	**0.08**	**80.5**	**36.8**	**0.012**
**12**	**29.5**	**18.1**	**48.4**	**24.8**	**0.001**	**28.7**	**18.1**	**0.30**	**47.3**	**35.4**	**0.284**
**13**	**30.1**	**14.4**	**42.2**	**30.6**	**0.041**	**43.8**	**34.4**	**0.33**	**63.1**	**44.2**	**0.115**
**14**	**28.7**	**12.9**	**47.9**	**29.6**	**0.001**	**41.4**	**27.7**	**0.67**	**57.2**	**21.9**	**0.028**
**15**	**28.5**	**12.8**	**38.7**	**23.3**	**0.026**	**38.2**	**22.6**	**0.78**	**62.2**	**35.7**	**0.142**
**16**	**27.6**	**12.0**	**37.6**	**26.6**	**0.045**	**37.2**	**22.0**	**0.88**	**51.7**	**37.5**	**0.171**
**17**	**27.1**	**12.1**	**38.2**	**23.8**	**0.016**	**33.6**	**25.5**	**0.79**	**49.4**	**20.5**	**0.014**
**18**	**29.1**	**15.6**	**31.7**	**21.6**	**0.56**	**36.1**	**19.9**	**0.87**	**32.3**	**12.0**	**0.142**
**19**	**29.7**	**17.3**	**32.6**	**16.0**	**0.47**	**35.6**	**18.0**	**0.79**	**50.5**	**28.9**	**0.310**
**20**	**35.6**	**18.9**	**42.4**	**21.2**	**0.16**	**43.4**	**18.5**	**0.24**	**78.5**	**25.4**	**0.007**
**21**	**48.2**	**22.8**	**55.9**	**33.7**	**0.26**	**57.3**	**32.3**	**0.54**	**108.7**	**44.0**	**0.009**
**22**	**55.1**	**24.7**	**63.0**	**36.6**	**0.29**	**63.7**	**23.4**	**0.84**	**127.7**	**42.0**	**0.006**
**23**	**62.2**	**26.1**	**68.5**	**28.6**	**0.33**	**70.0**	**35.2**	**0.53**	**137.1**	**39.9**	**0.001**
**24**	**62.8**	**25.1**	**72.9**	**31.5**	**0.14**	**81.4**	**37.2**	**0.52**	**145.1**	**76.3**	**0.019**
**1**	**58.9**	**29.0**	**70.2**	**36.9**	**0.16**	**76.2**	**38.4**	**0.86**	**95.9**	**29.1**	**0.009**
**2**	**62.1**	**30.4**	**68.1**	**35.0**	**0.44**	**71.7**	**45.2**	**0.51**	**114.0**	**29.9**	**0.040**
**3**	**67.1**	**34.6**	**67.3**	**33.4**	**0.98**	**78.4**	**31.3**	**0.77**	**93.1**	**32.6**	**0.026**
**4**	**69.8**	**28.8**	**70.8**	**31.1**	**0.88**	**68.2**	**28.6**	**0.31**	**99.8**	**73.1**	**0.296**
**5**	**59.5**	**25.8**	**58.0**	**27.3**	**0.81**	**60.4**	**29.2**	**0.20**	**75.2**	**16.3**	**0.125**
**6**	**60.2**	**28.2**	**55.3**	**30.0**	**0.48**	**53.5**	**20.3**	**0.17**	**87.3**	**40.0**	**0.173**
**7**	**44.8**	**23.2**	**43.9**	**21.6**	**0.87**	**32.4**	**12.8**	**0.06**	**53.0**	**29.7**	**0.823**

Hourly RMSSD values given as means (MW) and standard deviation (SD) of children with CGD, GHD and SGA during clonidine testing compared to baseline data of age-matched healthy controls. Bold types are indicating statistically significant differences between clonidine tested children and controls.

**Table 4:** HF/LF values during clonidine testing and healthy controls.

	Baseline		HF/LF during Clonidine Test started at 9:00 T- test versus age matched baseline controls								
	Healthy Control		Constitutional Growth Delay			Growth Hormone Deficiency			Small for Gestational Age		
	N = 56		N = 36			N = 14			N = 6		
Time	MW	SD	MW	SD	p-value	MW	SD	p-value	MW	SD	p-value
**8**	**0.52**	**0.34**	**0.49**	**0.26**	**0.71**	**0.38**	**0.27**	**0.19**	**0.38**	**0.13**	**0.83**
**9**	**0.44**	**0.45**	**0.59**	**0.55**	**0.22**	**0.50**	**0.33**	**0.57**	**0.99**	**0.97**	**0.25**
**10**	**0.38**	**0.23**	**0.68**	**0.41**	**<0.000**	**0.63**	**0.39**	**0.07**	**0.59**	**0.30**	**0.12**
**11**	**0.48**	**0.33**	**1.85**	**1.80**	**<0.000**	**1.71**	**1.96**	**0.024**	**0.94**	**0.90**	**0.17**
**12**	**0.49**	**0.33**	**1.37**	**1.20**	**<0.000**	**0.57**	**0.49**	**0.80**	**0.53**	**0.38**	**0.52**
**13**	**0.67**	**0.52**	**1.81**	**3.13**	**0.046**	**1.77**	**2.60**	**0.08**	**0.68**	**0.34**	**0.29**
**14**	**0.57**	**0.35**	**1.25**	**1.13**	**0.001**	**1.57**	**2.57**	**0.12**	**0.48**	**0.24**	**0.39**
**15**	**0.55**	**0.38**	**1.12**	**1.17**	**0.009**	**0.98**	**0.82**	**0.07**	**0.69**	**0.38**	**0.92**
**16**	**0.59**	**0.45**	**0.78**	**0.89**	**0.27**	**0.90**	**0.86**	**0.20**	**0.57**	**0.35**	**0.94**
**17**	**0.47**	**0.29**	**0.81**	**0.87**	**0.027**	**0.58**	**0.41**	**0.49**	**0.52**	**0.18**	**0.33**
**18**	**0.46**	**0.22**	**0.53**	**0.38**	**0.36**	**0.53**	**0.26**	**0.23**	**0.50**	**0.27**	**0.20**
**19**	**0.44**	**0.23**	**0.54**	**0.27**	**0.09**	**0.66**	**0.40**	**0.033**	**1.89**	**2.88**	**0.28**
**20**	**0.65**	**0.59**	**1.06**	**1.56**	**0.15**	**0.76**	**0.51**	**0.44**	**1.02**	**1.39**	**0.39**
**21**	**1.56**	**1.68**	**1.06**	**1.04**	**0.13**	**0.81**	**0.63**	**0.37**	**0.58**	**0.82**	**0.12**
**22**	**1.83**	**1.75**	**1.95**	**2.16**	**0.80**	**1.27**	**0.76**	**0.47**	**0.49**	**0.26**	**0.021**
**23**	**1.89**	**1.64**	**1.34**	**1.13**	**0.10**	**1.10**	**0.61**	**0.17**	**0.29**	**0.27**	**0.07**
**24**	**1.67**	**1.52**	**2.25**	**6.03**	**0.58**	**1.41**	**2.00**	**0.71**	**0.50**	**0.62**	**0.21**
**1**	**1.28**	**1.10**	**1.21**	**1.21**	**0.80**	**1.19**	**0.86**	**0.49**	**0.69**	**0.61**	**0.13**
**2**	**1.40**	**1.57**	**1.32**	**1.06**	**0.78**	**1.74**	**2.19**	**0.27**	**0.41**	**0.35**	**0.20**
**3**	**1.68**	**2.59**	**1.15**	**1.18**	**0.26**	**0.93**	**1.00**	**0.51**	**0.57**	**0.45**	**0.29**
**4**	**1.03**	**1.14**	**1.37**	**1.74**	**0.33**	**2.08**	**3.71**	**0.20**	**0.52**	**0.61**	**0.24**
**5**	**0.98**	**0.92**	**0.87**	**0.49**	**0.53**	**1.02**	**0.63**	**0.24**	**1.48**	**1.75**	**0.41**
**6**	**0.91**	**0.71**	**0.97**	**0.82**	**0.75**	**0.65**	**0.38**	**0.64**	**1.07**	**1.27**	**0.76**
**7**	**0.90**	**0.99**	**0.66**	**0.70**	**0.26**	**0.62**	**0.53**	**0.98**	**0.71**	**0.98**	**0.96**

Hourly HF/LF values given as means (MW) and standard deviation (SD) of children with CGD, GHD and SGA during clonidine testing compared to baseline data of age-matched healthy controls.

#### Correlation of IGF-1 and IGF- BP3 with HRV

Groups of short patients did not differ significantly regarding IGF-1 (insuline like growth factor) and IGF-BP3 (Insuline like binding protein). However analysing growth factors as IGF-1 and IGF-BP3 in relation to HRV we found significant correlations of global heart rate variability SDNN with IGF-1 (pearson r = 0.34**;p = 0.009) and IGF-BP3 (pearson r = 0.32*;p = 0.01).

## Discussion

To increase patient safety and to investigate the autonomic reaction after administration of a strong stimulus we measured HRV in pre pubertal children with growth failure during a routine clonidine growth hormone stimulation test. Only children with constitutional growth delay showed the expected increase of HRV, namely rMSSD, after clonidine administration and were considered as autonomous healthy. The children with GHD showed no changes of HRV parameter rMSSD after clonidine administration as a sign of an underlying hypothalamic problem (Fig 1). These results indicate that at least these children with GHD may have a genuine sub sensitivity of the α_2_-adrenoreceptor and growth hormone deficiency in this case is not caused by a pituitary problem. However the small group of children with SGA without catch up growth showed only slight vagal activation by means of increasing rMSSD but no sympathetic inhibition by means of HF/LF increase after clonidine administration (Fig 2). The role of low IGF1 levels in SGA patients remains unclear, but our results are consistent with data of Albertsson-Wikland et al. [15] and Boguszewski et al. [16]. Groups of CGD, GHD and SGA did not differ significantly in IGF-1 and IGF-BP3 levels. But analyzing these growth factors for correlation with HRV we found rather convincing correlations for IGF-1 stronger than for IGF-BP3 with global heart rate variability SDNN. Keeping in mind that low levels of these growth factors are diagnostic criteria for GHD and are well described for SGA we account these findings to the here described impairment of the central α_2_-adrenoreceptor.

Analysing children with growth deficiency showed that height was significantly reduced compared with normal controls (p < .001), so was body weight (p < .01 for SGA and GHD, p < .001 for CGD patients) (Table 1). Realizing that body mass index (BMI) did not differ from healthy controls, normal nutritional status can be assumed in our patients. According to data from Khadilkar et al. addressing body composition [17] we have to assume a higher amount of fat mass related to lean mass in GHD children. However assessing possible influences on HRV we rather suggest to focus on nutritional stage than on body composition. In our recently published work [18] we describe the influence of nutritional state on autonomic regulation addressing adiposity and starvation. Since we find normal nutritional status in the patients described here, influence of nutritional state on autonomic regulation in this case seems to be unlikely.

Focussing on night time HRV of our patients we recognize in the SGA group a significant increase of rMSSD between 20:00 and 03:00 o clock at night (p .007- .04) (Fig 1, Table 2). In order to clarify this finding rMSSD data was compared to an additional age-matched control of SGA children, who did not undergo clonidine testing. SGA without clonidine testing showed normal rMSSD compared to the control group (Fig 3).This finding might be interpreted as a late reaction of the central α_2_-adrenoreceptor to clonidine representing a different pattern of autonomic regulation in this group. However taking into account the rather small group of SGA patients in our study there might even be a statistical bias, which we consider to be more likely.

The Framingham Heart study showed that autonomic dysregulation is present in the early stage of hypertension [19]. Therefore it has to be assumed that these disturbances of the autonomic nervous system are early surrogate parameters for the enhanced cardiovascular risk in some patients with short stature. Based on this concept growth hormone therapy eases the lack of growth hormone and provides normalised longitudinal growth. The impaired sensitivity of the central α_2_-adrenoreceptor remains untreated. Whether there is an additional increase of cardiovascular risk by recombinant growth hormone remains topic of future research.

Our results are in accordance with the current literature [20,21], that shows significantly reduced HRV in children with a birth weight below 2500g now aged between 9 and 10 years. In contrast HRV parameters were significantly enhanced in 15 healthy adults aged 20 to 30 years with low birth weight <2500g published by G. Weitz et al. [22]. However a similar height compared to the control group shows that-in contrast to our data- in this study only SGA patients with catch up growth were analysed. Similar to this finding sympathetical activity was reduced in fourteen untreated adults with GHD at mean age 35.2years [23].

Growth hormone replacement therapy seems to increase sympathetic tone. This finding is interpreted as normalization of HRV by Leong et al. as well as Tanriverdi et al. [24,25]. Taking into account our findings of reduced vagal activation after stimulation of the central α_2_-adrenoreceptor the results of adult HRV may represent a developmental adaptation of the adult autonomic system. However compared to normal values of Bonnemeier et al. [26] baseline values of GHD adults from the Leong group [23] seem to be rather in normal range. From this point of view increasing sympathetic tone should rather be interpreted as an early sign for increasing cardiovascular risk. The effect of growth hormone replacement therapy on HRV in adults with GHD still remains unclear.

Little is known of the long term health of children treated with growth hormone in childhood. SAGhE is a European consortium targeting this question. A preliminary report of the French SAGhE study group showed an increase in mortality in 6928 children due to disease of the circulatory system (SMR 3.07, 95% CI 1.4–5.83) [27]. This excess mortality is related to the highest growth hormone doses >50 μg/kg/day (SMR 2.94, 95% CI 1.22–7.07) but also to height at initiation of treatment <−3 SDS (SMR 2.31, 95% CI 0.96–5.59). Consolidated results of the complete SAGhE study were expected to be disseminated during 2013 (still missing) but it will be the fate of this high volume project not to be able to differentiate the expected excess cardiovascular mortality due to short stature from the treatment effect of growth hormone therapy. However, this important question has to be answered regarding approximately 40000 children being treated in Europe and several hundred thousand in the world. By using a conclusive pathophysiological model as shown in this paper it will be possible to evaluate the cardiovascular safety of growth hormone therapy.

In contrast to constitutional growth delay, idiopathic isolated growth hormone deficiency and small for gestational age seem to display attributes of a “stress” disease independent from actual psychosocial stress due to short stature, which is equal in these groups. A functional α_2_-adrenoreceptor sub sensitivity mediating clonidine induced growth hormone secretion has been described in Wistar rats after chronic hydrocortisone treatment simulating stress [28]. A link between diurnal cortisol rhythms and child growth was shown in a longitudinal study exploring the life history consequences of hypothalamic pituitary axis in children of a foraging society in the Bolivian Amazon [29].

**Fig 4:**
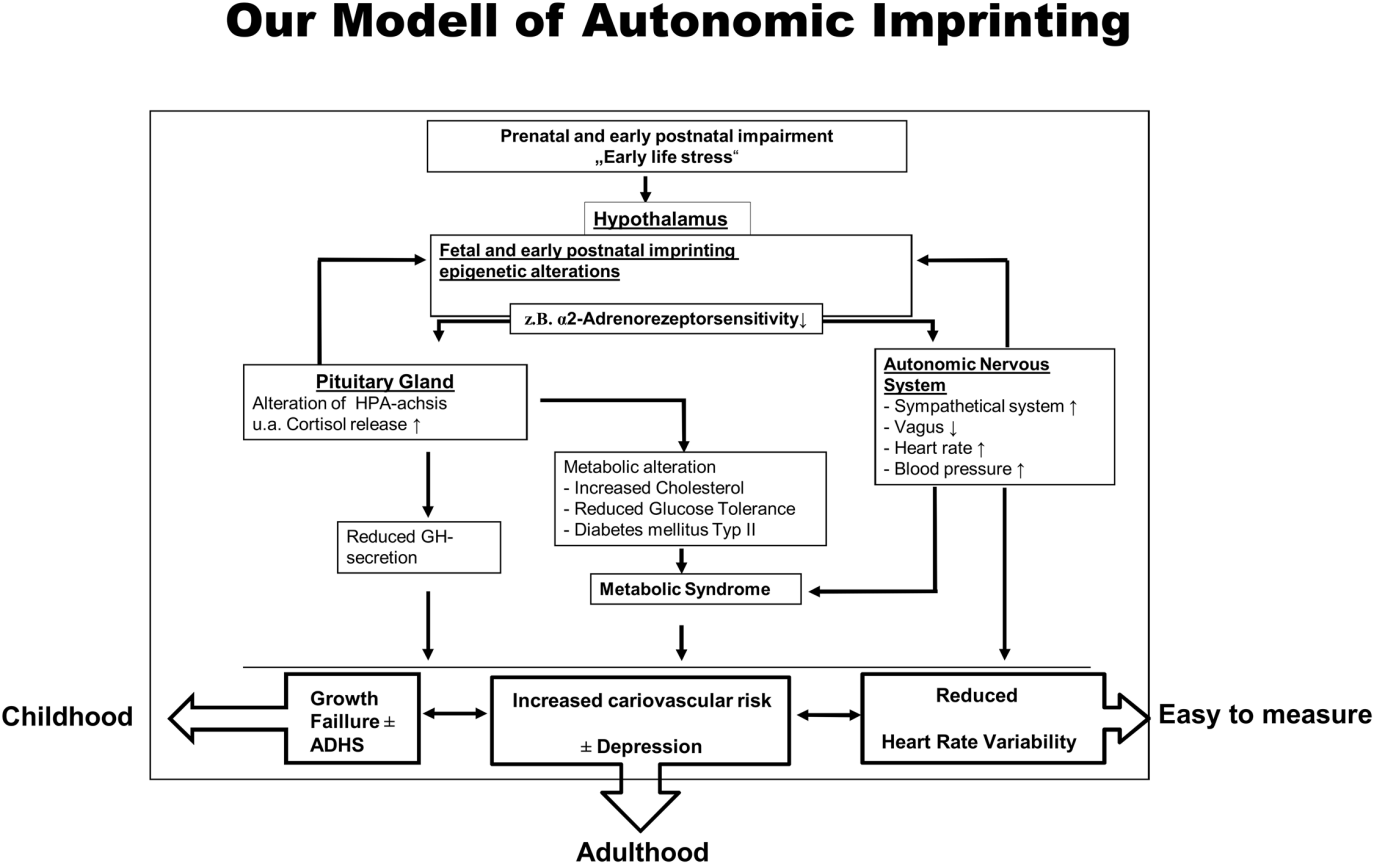
Our model of autonomic imprinting. Early life stress during pregnancy or short time after birth imprints the hypothalamic α_2_-adrenoreceptor. Depending on its regulatory properties the pituitary axis as well as the autonomic nervous system are altered resulting in growth delay, altered adrenal function and reduced HRV.

We speculate that hypothalamic α_2_-adrenoreceptor sub sensitivity as shown in children with GHD and probably in SGA is the missing link between early programming of the hypothalamic pituitary axis, growth failure and enhanced cardiovascular risk [30,31]. Our model about how early life stress may cause stunting, enhanced cardiovascular risk and impaired neurodevelopment is illustrated in Fig 4. In the centre of our reflections stands the hypothalamic α_2_-adrenoceptor, which seems to play an important role in the regulation of the hypothalamus-pituitary-axis and the autonomic nervous system. A sub sensitivity of hypothalamic α_2_-adrenoceptor, as shown in children with growth hormone deficiency and small for gestational age, may explain many symptoms of these children including stunting, enhanced cardiovascular risk and neurocognitive disorders. According to this idea recently published data show that reduced heart rate variability is adversely associated with metabolic syndrome [32,33]. Understanding these mechanisms, whereby early stressors may be transmitted from mother to foetus, will not only improve our knowledge of normal foetal and neonatal development but will also help to identify novel pathways for early interventions.

Current practice of growth hormone stimulation testing is impaired by the difficulty in putting up cut off values [34] as well as by remarkable high between- assay variability of the available immunoassays [35]. Adding the idea of sub sensitivity of the hypothalamic α_2_-adrenoreceptor to diagnosing GHD may enrich the difficult interpretation of growth hormone stimulation testing. Reduced autonomic response after clonidine testing as described here may be an additional criterion for diagnosing growth hormone deficiency.

Our simple modification of the clonidine growth hormone stimulation test by simultaneous HRV analysis may help us to examine the function of the hypothalamic α_2_-adrenoceptor in different somatic and psychosomatic diseases. As shown in children with GHD, we are able to detect α_2_-adrenoreceptor sub sensitivity a long time before global HRV may be reduced. The test may be able to analyse the hypothalamic α_2_-adrenoceptor function not only in patient groups but also on an individual base if we use 24-hour HRV a day before the clonidine test as individual baseline.

### Study limitations

One clear limitation of our study is the rather small sample size especially the small number of SGA. However the idea of investigating the reaction of HRV attributed to a strong stimulus as clonidine within clearly defined groups of patients strengthens the quality of data. Another limitation is the design not being prospective. For data analysis all consecutive patients within the study period, who met criteria have been included. To proof the outlined concept of reduced α_2_-adrenoreceptor sub sensitivity as a pathophysiological agent in children with isolated growth hormone deficiency further prospective research will be necessary.
