# Correction: Modified Clonidine Testing for Growth Hormone Stimulation Reveals α_2_-Adrenoreceptor Sub Sensitivity in Children with Idiopathic Growth Hormone Deficiency

**DOI:** 10.1371/journal.pone.0141291

**Published:** 2015-10-20

**Authors:** Christian Willaschek, Sebastian Meint, Klaus Rager, Reiner Buchhorn

There are errors in Table 2. Please see the corrected Table 2 here.

**Table:** 

		Clonidine Test (Age matched T-Tests)		
Parameter	Healthy Control	Constitutional Growth Delay	Growth Hormone Deficiency	Small for Gestational Age
**N**	56	36	14	6
**IgF1 [100–476 ng/ml]**		86 ± 56	76 ± 42	43 ± 30
**IgFPB3 [1900–5200 μg/l]**		3075 ± 1076	2863 ± 1254	2538 ± 644
**NT-pro-BNP [<289 ng/l]**		162 ± 169	178 ± 172	177 ± 94
**Heart Rate [bpm]**	90 ± 15	85 ± 11***	85 ± 11	83 ± 8*
**SDNN [ms]**	149 ± 51	112 ± 27**	117 ± 33*	142 ± 40
**RMSSD[ms]**	46 ± 18	51 ± 19*	50 ± 17	77 ± 15**
**RMSSD Day**	34 ± 15	41 ± 16**	37 ± 16	53 ± 14**
**RMSSD Night**	61 ± 26	65 ± 24	70 ± 24	108 ± 24**
**Total Power**	4957 ± 3029	3992 ± 2082	4000 ± 1873	7298 ± 2580**
**Total Power Day**	3889 ± 2747	3048 ± 1629	3286 ± 1863	4720 ± 1971*
**Total Power Night**	6287 ± 3843	5289 ± 3118	5111 ± 2391	10740 ± 3264**
**VLF**	2839 ± 2222	1960 ± 1327	2026 ± 1085	4831 ± 2678*
**VLF Day**	2256 ± 2016	743 ± 124	1666 ± 947	2710 ± 1601
**VLF Night**	3534 ± 2844	2699 ± 2412	2581 ± 1595	7553 ± 3833*
**LFn**	60 ± 8	52 ± 10*	55 ± 10	65 ± 12
**LFn Day**	65 ± 9	53 ± 9***	59 ± 11	61 ± 12
**LFn Night**	56 ± 10,6	51 ± 14	53 ± 12	70,8 ± 13
**HF**	731 ± 350	817 ± 334	759 ± 295	747 ± 376
**HF Day**	456 ± 271	636 ± 371**	553 ± 331	633 ± 268*
**HF Night**	1100 ± 556	1082 ± 451	1079 ± 483	945 ± 671
**HF/LF**	0.61 ± 0.22	0,85 ± 0,36*	0,78 ± 0,32	0,51 ± 0,33
**HF/LF Day**	0,45 ± 0,18	0,77 ± 0,34***	0,65 ± 0,32	0,59 ± 0,40
**HF/LF Night**	0,79 ± 0,38	0,99 ± 0,55	0,95 ± 0,66	0,43 ± 0,30*

Laboratory data, data on 24- hour HRV, daytime und night time HRV from children with CGD, GHD and SGA compared to age-matched healthy controls.**IgF1:** Insuline like growth factor, **IgFPB3:** Insuline like growth factor binding proteine, **NT-pro-BNP:** N-terminal propeptide of brain natriuretic peptide, **SDNN:** Standard deviation of NN-intervalls, **RMSSD:** Root mean square standard deviation of adjacent NN Intervals, **VLF:** Very low frequency (< 0.04 Hz),**LF:** low frequency (LF, 0.04–0.15 Hz) **HF:** high frequency (HF, 0.15–0.4 Hz)

*** p < 0.001

** p < 0.01

* p < 0.5
